# Bone mineral density in young adults: the influence of vitamin D status, biochemical indicators, physical activity and body composition

**DOI:** 10.1007/s11657-020-0684-0

**Published:** 2020-03-12

**Authors:** Anna Kopiczko, Monika Łopuszańska-Dawid, Karol Gryko

**Affiliations:** 1 Department of Biomedical Sciences, Józef Piłsudski University of Physical Education in Warsaw, Marymoncka 34, 00-968 Warsaw, Poland; 2 Department of Individual and Team Sports, Józef Piłsudski University of Physical Education in Warsaw, Marymoncka 34, 00-968 Warsaw, Poland

**Keywords:** Mineralised forearm bones, Body fat and lean body mass, Phosphorus, Magnesium, Lipid profile, Physical activity

## Abstract

***Summary*:**

The aim of the study was to assess the associations of bone mineral density and bone mass with physical activity levels, vitamin D, phosphorus, magnesium, total cholesterol and triglyceride concentration and body composition in young women and men. Physical activity has the most significant effect on bone status especially in men.

**Purpose:**

The aim of the study was to assess the associations of bone mineral density and bone mass with physical activity levels, vitamin D, phosphorus, magnesium, total cholesterol and triglyceride concentration and body composition in young women and men.

**Methods:**

One hundred subjects aged 19–24 years were included. Bone mineral density (BMD) in distal and proximal parts was evaluated by forearm densitometry. Body composition was analysed with the use of JAWON-Medical-x-scan. The following biochemical indicators were analysed: 25(OH) D and 1,25(OH)2D, magnesium, phosphorus, total cholesterol and triglycerides. Physical activity levels were assessed by interview.

**Results:**

Significant correlations between BMD and physical activity, skeletal muscle mass and body fat percentage were revealed in men. Among women, considerably weaker correlations of BMD with body composition and physical activity were noted than in men. BMD in the distal part correlated only with lean body mass, soft lean mass and body fat percentage. The strongest relationship between physical activity and bone mineral status parameters was noted for BMD in men. In women, physical activity did not affect BMD.

**Conclusions:**

Physical activity has the most significant effect on bone status especially in men.

## Introduction

The state of bone tissue as well as an excessive loss of bone mass diagnosed in young people more and more often constitutes a significant public health problem. Risk factors of osteopenia and, as a consequence, of osteoporosis occur mainly as a result of unbalanced diet and unhealthy lifestyle and, first and foremost, as a result of insufficient physical activity [1]. Proper nutrition with particular consideration of calcium, protein, phosphorus and magnesium intake as well as appropriate sun exposure ensuring endogenous synthesis of vitamin D exerts a huge influence on the correct bone development [2, 3].

Vitamin D in the form of 1,25(OH)2D is a steroid hormone. Serum 25(OH) D concentration constitutes an indicator of vitamin D level in the body. Biologically active vitamin D metabolite, i.e. 1,25(OH)2D, is one of the main factors regulating metabolism of calcium and phosphorus. Moreover, it was revealed that 1,25(OH)2D directly and indirectly affects proteins which are crucial for osseous metabolism and controls their synthesis at every stage of osteoblast differentiation and bone remodelling [4, 5]. An optimal level of vitamin D is important for good health and marking its active metabolites in serum reflects its supply from diet and photosynthesis in the skin. Recently, an interest in vitamin D has increased considerably among physically active individuals who are recommended to take vitamin D on everyday basis. This interest in vitamin D is caused not only by the prevention of bone diseases connected with an insufficient level of vitamin D but also by the fact that vitamin D receptors were discovered in many tissues, which implies a complex role of vitamin D particularly in young people. The population-based study revealed alarmingly low levels of 25(OH) D in serum (< 10 ng/mL in approximately 30% of the population, < 20 ng/mL in approximately 80% of the population), which may indicate a low vitamin D supply in the European population, particularly in winter [6–8].

Other elements of a diet also exert a significant influence on the state of bones. Hydroxylapatite, which is a bone mineral, is made of calcium and phosphorus whose appropriate intake may be important for bone health [9, 10]. Research on rats revealed that, in a group with a diet high in phosphorus, BMD dropped significantly compared with the control group [11]. In a randomised study, a high phosphorus intake negatively affected bone metabolism in healthy women by increasing bone resorption and reducing bone development. Phosphorus is significant for bone creation but its excessive intake increases the level of phosphates in serum and disturbs hormonal regulation of calcium and phosphorus. It may lead to lower bone endurance and may increase the risk of fractures. Harmful effects of excessive phosphorus intake on the development of bones are noted when calcium intake is low [12, 13].

Some studies point to important relationship between the state of bone tissue and the lipid level in the blood serum. Lipid disorders and their relation to a low mineral density of the bones are defined as the multifactorial process. The mechanism of this action can be directly related to the biosynthetic pathway of cholesterol [14]. Some studies have noted a negative correlation between the atherogenic lipid profile and BMD and other studies showed no relationship between them [14, 15].

The influence of physical activity and loads related to it on bone tissue needs further research and more detailed analysis. Previous observations revealed that resistance exercises which generate appropriate forces on bone tissue ensure good trophic of this tissue and support its proper development. High-intensity power training is proved to provide substantial improvement for the hip, trochanter and lumbar spine BMD [16]. Body vibration in complex aerobic and resistance training programs also increases lumbar BMD [17].

On the other hand, several studies revealed that the protective influence of physical activity on bone tissue may be defined as the local one. It was proved that physical activity is one of the factors which lead to achieving high peak bone mass and reducing its age-related loss. Physical exercises, mainly resistance ones, locally affect the bone system, thus leading to better mechanical endurance of bones. According to the ‘Mechanostat theory’, the consolidation of the skeleton results from mechanical adaptation of bone tissue to increasing loads generated by muscle tissue [18, 19].

The recommended activities include exercises during which body mass withstands gravity. Numerous cross-sectional studies indicated positive effects of weight-bearing activity [20, 21]. The results of observational studies showed that physically active individuals had a higher BMD and were at a lower risk of bone fracture occurring in the course of osteoporosis development than the study participants with a sedentary lifestyle. This correlation may depend on a higher percentage of muscle mass in the body composition of active individuals resulting from physical activity. Pressure put by active muscles on bone tissue is necessary for its appropriate development [22, 23].

The aim of the study was to assess the associations of bone mineral density and bone mass with physical activity levels, vitamin D, phosphorus, magnesium, total cholesterol and triglyceride concentration and body composition in young women and men.

## Methods

### Sample and procedure

This research included 100 individuals, i.e. 50 women (age 22.9 ± 3.6 years) and 50 men (age 23.4 ± 4.4 years). The selection of the sample from the population list of the Polish capital was designed as part of a scientific research (DS. 272/2018/AWF). The research sample was drawn several times and systematically selected until the planned number of 100 examined people meeting the inclusion criteria was obtained. The average reporting rate for similar screening tests in Poland is around 25–30% [24].

The inclusion criteria were as follows: written consent to participate in the study and their permission for venous blood draw as well as the lack of health contraindications to densitometry and body composition analysis. The work described has been carried out in accordance with The Code of Ethics of the World Medical Association (Declaration of Helsinki) for experiments involving humans. The project was approved by the Research Ethics Committee (number SKE 01-09/2017).

Physical activity levels were assessed in a direct interview regarding the participation in sports and recreational activities as well as habitual physical activity. Both gender groups were divided into individuals who were physically active (60% of the women, 65% of the men) and physically inactive individuals who did not take up any additional physical activity (40% of the women, 35% of the men). The physically active group was characterised by daily physical activity over 30 min and systematic participation by practicing various team sports (basketball, volleyball, handball) 4–5 times a week for 45–60 min.

### Measurements

Vitamin D and its active metabolite were assessed on the basis of 25-hydroxyvitamin D [25(OH) D] and 1,25-dihydroxyvitamin D [1,25(OH)2D] concentration in serum (in ng/mL) with chemiluminescent immunoassay (CLIA) using the IDS-iSYS analyser certified by the Vitamin D External Quality Assessment Scheme (DEQAS). Concentration levels were marked according to medical procedures. Magnesium (mmol/L) and phosphorus (mmol/L) were also assessed on the basis of their concentration levels in serum. In order to determine biochemical indicators, blood samples were taken from the elbow vein at rest in the morning on an empty stomach. Densitometry, anthropometric measurements and body composition tests were performed the following day. Total cholesterol concentration (TC mmol/L) and triglyceride concentration (TG mmol/L) in serum were determined with the use of diagnostic kits.

Bone mineral density (BMD), bone mass content (BMC) and T-scores of non-dominant forearm were measured by means of the dual-energy X-ray absorptiometry (pDEXA) method using Norland instrument. The effective dose (μSv) for this densitometer is 0.05. The length of the forearm was measured using large anthropometry callipers at the radiale-stylion points (r-sty). There were two measurement points: at the proximal and the distal parts of bone according to the adopted method of densitometry. The Norland pDEXA has a general distal site (radius + ulna), a general ^1^/_3_ proximal site (radius + ulna) and a ^1^/_3_ proximal radius site. Regression statistics were reported for all similar regions of interest (ROIs). The distal ROIs span 10 mm of the lowest BMD region in the distal forearm and are found using an automated search routine. The proximal site spans 10 mm starting at the ^1^/_3_ forearm length and continuing proximally [25]. Body composition was analysed with the use of bioelectrical impedance analysis device JAWON-Medical x-scan. The levels of lean body mass (LBM, kg), soft lean mass (SLM, kg), skeleton muscle mass (SMM, kg) and body fat percentage (BFP, %) were assessed. Basic body dimensions and indices were evaluated with the use of anthropometric measurements. Body height, body mass and waist and hip circumferences as well as non-dominating forearm length were measured. Body mass index (BMI) was also calculated.

### Statistical analysis

The research results were analysed with the use of Statistica software (v.11, Stat. Soft., USA). In order to determine the significance of differences between the values of particular variables for men and women, Student’s *t* test for independent variables was applied. The same test was also used to assess differences between variables in the distal and proximal parts in the groups of men and women. Differences between the frequency of occurrence of appropriate and decreased bone mineralisation as well as proper and improper concentrations of the analysed biomarkers in serum were analysed with the use of the chi-square test. The ANCOVA was applied in order to find relationships between bone mineral density and body composition, concentration of selected biochemical indicators, lipid profile and physical activity (as qualitative predictor). In turn, the ANOVA with Tukey’s post hoc test was used to evaluate significance of differences in bone mass in the context of physical activity. Statistical significance was set at the levels of *p ≤ 0.05, **p ≤ 0.01 and ***p ≤ 0.001.

## Results

Table 1 presents the frequency of occurrence of proper and decreased bone mineral status as well as proper and improper concentrations of the analysed biochemical indicators in men and women. Decreased BMD was noted mainly in the proximal part in nearly half of the men and over half of the women. The evaluation of the frequency of deficits and improper concentration of the analysed biochemical indicators revealed 25(OH) D deficit in over half of the men and in nearly one in four women. The desired value of TC occurred significantly more often in men (by 22%), while a high border level was noted more often in women (Table 1).

**Table 1:** The frequency of occurrence of proper and decreased bone mineral status as well as proper and improper concentrations of the analysed biochemical indicators in men and women (%) and percentage differences (chi^2^ test, level of significance *p*)

	Reference ranges	Men, %	Women, %	Chi^2^ (*p*)
Bone mineral density				
Distal part	Low BMD (T-score ≤ − 1.00)	4	2	0.3501 (0.5541)
	Normal BMD (T-score > − 0.99)	96	98	
Proximal part	Low BMD (T-score ≤ − 1.00)	46	56	1.0021 (0.3168)
	Normal BMD (T-score > − 0.99)	54	44	
Biochemical indicators				
25(OH) D (ng/mL)	Deficit 0–20 ng/mL	52	38	2.0634 (0.3564)
	Sub-optimal concentration > 20–30	34	46	
	Optimal concentration > 30–50	14	16	
1,25(OH)2D (pg/mL)	Deficit < 20 pg/mL	8	6	1.5296 (0.4654)
	Proper concentration 20–60 pg/mL	92	92	
	Higher concentration	0	2	
Phosphorus (mmol/L)	Deficit < 0.81 mmol/L	0	0	1.3964 (0.2373)
	Proper concentration 0.81–1.62 mmol/L	98	100	
	Too high concentration > 1.62 mmol/L	2	0	
Magnesium (mmol/L)	Deficit < 0.65 mmol/L	4	16	5.7923 (0.0552)
	Proper concentration 0.65–1.2 mmol/L	96	82	
	Too high concentration > 1.2 mmol/L	0	2	
Lipid profile	Normal < 1.7 mmol/L	90	86	0.3804 (0.5374)
TG (mmol/L)	Border high 1.7–2.3 mmol/L	10	14	
TC (mmol/L)	Desired < 5.2 mmol/L	84	62	6.2784 (0.0122)*
	Border high 5.2–6.1 mmol/l	12	34	
	High ≥ 6.2 mmol/L	4	4	

Statistical significance was set at the levels of *p ≤ 0.05

The analysis of particular variables in the groups of normal and decreased bone mass in men revealed significantly higher (*p* ≤ 0.001) BMI values and body composition elements such as LBM, SLM and SMM in the participants with normal BMD in the proximal part compared with the individuals with decreased BMD in the same part. Bone mineralisation was not affected by biochemical indicator concentration levels (Table 2).

**Table 2:** Means and standard deviations of particular variables in the groups of normal and lower bone mass for distal and proximal parts separately as well as significance of differences between the means (Student’s *t* test, *p*) in *men*

	Bone mineral density (g/cm^2^)			
	Distal part		Proximal part	
	Normal BMD, mean (SD)	Low BMD, mean (SD)	Normal BMD, mean (SD)	Low BMD, mean (SD)
Body composition				
BMI (kg/m^2^)	24.5693 (2.3519)	21.7547 (0.2300)	25.3027 (2.3458)	23.4636 (2.0243)
*t* (*p*)	2.8078 (0.1003)		8.6450 (0.0050)*	
LBM (kg)	67.8771 (8.4061)	66.5500 (0.6364)	71.8444 (7.5122)	63.1043 (6.4273)
*t* (*p*)	0.0489 (0.8260)		19.1660 (0.0001)**	
SLM (kg)	63.3292 (7.8727)	62.1000 (0.4243)	66.8185 (7.0732)	59.1261 (6.3260)
*t* (*p*)	0.0478 (0.8279)		16.1730 (0.0002)**	
SMM (kg)	34.7063 (6.9252)	33.0500 (1.7678)	38.1037 (6.3211)	30.5739 (4.8588)
*t* (*p*)	0.1120 (0.7393)		21.6920 (0.0001)**	
BFP (%)	16.4229 (5.0793)	11.7500 (2.1920)	16.0852 (5.0798)	16.4130 (5.1656)
*t* (*p*)	1.6531 (0.2047)		0.0509 (0.8224)	
Biochemical indicators				
25(OH) D (ng/mL)	21.0500 (7.0132)	18.3000 (2.6870)	21.8778 (6.9647)	19.8391 (6.8113)
*t* (*p*)	0.3006 (0.5861)		1.0858 (0.3026)	
1,25(OH)2D (pg/mL)	45.4104 (14.8440)	43.75 (6.2933)	44.3593 (14.5182)	46.5000 (14.8689)
*t* (*p*)	0.0244 (0.8764)		0.2641 (0.6067)	
Phosphorus (mmol/L)	1.2192 (0.1657)	1.0050 (0.0071)	1.2348 (0.1415)	1.1822 (0.1936)
*t* (*p*)	3.2743 (0.0766)		1.2290 (0.2732)	
Magnesium (mmol/L)	0.8748 (0.1545)	0.7150 (0.0354)	0.8533 (0.1774)	0.8861 (0.1244)
*t* (*p*)	2.0961 (0.1542)		0.5520 (0.4611)	
Lipid profile				
TG (mmol/L)	1.0779 (0.5035)	1.0800 (0.0849)	1.0715 (0.5151)	1.0857 (0.4778)
*t* (*p*)	0.0001 (0.9954)		0.0100 (0.9206)	
TC (mmol/L)	4.5675 (0.7676)	4.3100 (0.2970)	4.6837 (0.7986)	4.4087 (0.6872)
*t* (*p*)	0.2200 (0.6412)		1.6720 (0.2022)	

Statistical significance was set at the levels of *p ≤ 0.05 and **p ≤ 0.01

Analogous calculations were made for women. Significantly higher values (*p* ≤ 0.01) of LBM, SLM and SMM were noted in women with normal BMD in the proximal part than in the participants with decreased BMD. In turn, in the distal part of the forearm, women with normal BMD demonstrated significantly higher phosphorus concentration than women with decreased BMD. The remaining biochemical indicator concentration levels in the groups of women with different bone statuses did not differ significantly (Table 3).

**Table 3:** Means and standard deviations of particular variables in the groups of normal and lower bone mass for distal and proximal parts separately as well as significance of differences between the means (Student’s *t* test, *p*) in *women*

	Bone mineral density (g/cm^2^)			
	Distal part		Proximal part	
	Normal BMD, mean (SD)	Low BMD, mean (SD)	Normal BMD, mean (SD)	Low BMD, mean (SD)
Body composition				
BMI (kg/m^2^)	21.5302 (2.23588)	20.3816 (−)	22.0161 (2.1904)	21.1075 (2.1971)
*t* (*p*)	0.2587 (0.6134)		2.1120 (0.1526)	
LBM (kg)	46.3653 (5.0049)	48.4000 (−)	48.4636 (5.0063)	44.7893 (4.3621)
*t* (*p*)	0.1620 (0.6891)		7.6760 (0.0079)**	
SLM (kg)	42.8224 (4.5657)	44.8000 (−)	44.7000 (4.6028)	41.4179 (3.9760)
*t* (*p*)	0.1838 (0.6700)		7.3080 (0.0095)**	
SMM (kg)	21.4082 (4.0640)	22.8000 (−)	23.0364 (3.1069)	20.1786 (4.2659)
*t* (*p*)	0.1149 (0.7360)		6.9590 (0.0112)*	
BFP (%)	23.8388 (4.5575)	20.7000 (−)	24.0773 (4.3881)	23.5393 (4.7090)
*t* (*p*)	0.4648 (0.4987)		0.1710 (0.6814)	
Biochemical indicators				
25(OH) D (ng/mL)	22.8204 (8.2645)	18.0000 (−)	21.8778 (6.9647)	19.8391 (6.8113)
*t* (*p*)	00.3334 (0.5664)		1.0858 (0.3026)	
1,25(OH)2D (pg/mL)	49.4531 (18.6019)	36.6000 (−)	44.3593 (14.5182)	46.5000 (14.8689)
*t* (*p*)	0.4679 (0.4973)		0.2641 (0.6067)	
Phosphorus (mmol/L)	1.2453 (0.1716)	0.8200 (−)	1.2468 (0.1799)	1.2289 (0.1832)
*t* (*p*)	6.0234 (0.0178)*		0.1190 (0.7313)	
Magnesium (mmol/L)	0.8492 (0.2090)	0.6800 (−)	0.8695 (0.2524)	0.8271 (0.16837)
*t* (*p*)	0.6420 (0.4270)		0.5055 (0.4805)	
Lipid profile				
TG (mmol/L)	0.9002 (0.4377)	0.8600 (−)	0.9523 (0.4173)	0.8579 (0.4485)
*t* (*p*)	0.0083 (0.9279)		0.5801 (0.4500)	
TC (mmol/L)	4.9629 (0.8607)	5.2300 (−)	5.1359 (0.6871)	4.8365 (0.9547)
*t* (*p*)	0.0944 (0.7600)		1.5360 (0.2212)	

Statistical significance was set at the levels of *p ≤ 0.05 and **p ≤ 0.01

The results of the covariance analyses between BMD and selected parameters (ANCOVA) indicated that, in the case of men, the main parameters affecting BMD in the distal part were triglycerides and body fat percentage (BFP). In turn, BMD in the proximal part was affected by body composition elements (SMM, BFP) as well as the level of 1,25(OH)2D (pg/mL) and physical activity. Similar analyses in women indicated a statistically significant (*p* ≤ 0.05) correlation only between physical activity and BMD in the distal part (Table 4).

**Table 4:** Relationships between BMD and body composition, biochemical indicators, lipid profile and physical activity levels in men and women (results of ANCOVA, *F* test, *p*)

	Bone mineral density (g/cm^2^)			
	Distal part		Proximal part	
	*F*	*p*	*F*	*p*
Men				
BMI (kg/m^2^)	1.6012	0.4800	3.3001	0.0712
LBM (kg)	0.1045	0.2146	1.0176	0.3178
SLM (kg)	0.4920	0.7503	1.4486	0.2330
SMM (kg)	4.6590	0.4889	7.2282**	0.0087
BFP (%)	5.7057*	0.0397	8.1906**	0.0059
25(OH) D (ng/mL)	1.2196	0.3850	2.9485	0.0920
1,25(OH)2D (pg/mL)	0.5023	0.2751	5.4796*	0.0215
Phosphorus (mmol/L)	0.7915	0.8163	0.7784	0.3823
Magnesium (mmol/L)	4.1436	0.3618	0.5720	0.4083
TG (mmol/L)	1.9295*	0.0179	1.3762	0.2401
TC (mmol/L)	0.1780	0.1703	0.1563	0.6952
Physical activity	2.6312	0.1412	7.2298**	0.0068
Women				
BMI (kg/m^2^)	0.7209	0.4129	0.0008	0.9816
LBM (kg)	0.0272	0.8619	2.4655	0.1222
SLM (kg)	0.0112	0.8963	2.4190	0.1202
SMM (kg)	0.0132	0.9134	1.2019	0.2799
BFP (%)	1.0171	0.3250	0.7637	0.3903
25(OH) D (ng/mL)	0.1435	0.7162	0.6596	0.4205
1,25(OH)2D (pg/mL)	0.3695	0.5500	1.0300	0.3115
Phosphorus (mmol/L)	0.0096	0.9933	0.0008	0.9804
Magnesium (mmol/L)	0.6193	0.4285	0.0672	0.8006
TG (mmol/L)	1.1550	0.2995	0.9029	0.3450
TC (mmol/L)	0.1433	0.7162	0.0698	0.7909
Physical activity	5.0857*	0.0203	0.6513	0.4199

Statistical significance was set at the levels of *p ≤ 0.05 and **p ≤ 0.01

The results of the analyses of correlations between BMC and selected variables indicated that BMC in the distal part correlated significantly with physical activity. Physically active men demonstrated significantly higher BMC in the distal part than physically inactive individuals. BMC in the proximal part in men correlated significantly with physical activity, SMM and BFP. Similar to BMD, the analyses of correlations of BMC with selected parameters in women turned out to be weaker than in men. BMC in the distal part correlated significantly only with body composition elements (LBM, SLM, BFP; *p* ≤ 0.05). A weak correlation between BMC and magnesium concentration in serum was noted in the proximal part in women (Table 5).

**Table 5:** Relationships between BMC and body composition, biochemical indicators, lipid profile and physical activity levels in men and women (results of ANCOVA, *F* test, *p*)

	Bone mineral content (g)			
	Distal part		Proximal part	
	*F*	*p*	*F*	*p*
Men				
BMI (kg/m^2^)	0.5212	0.4712	3.2895	0.0752
LBM (kg)	0.4688	0.4998	0.0174	0.8789
SLM (kg)	0.5089	0.4799	0.1303	0.7143
SMM (kg)	1.7863	0.1701	6.6668**	0.0121
BFP (%)	1.8364	0.1823	8.5432**	0.0048
25(OH) D (ng/mL)	0.4975	0.4850	0.7426	0.3903
1,25(OH)2D (pg/mL)	0.8219	0.3715	2.4956	0.1159
Phosphorus (mmol/L)	0.0855	0.7820	0.1507	0.7000
Magnesium (mmol/L)	0.5108	0.4701	0.0502	0.8386
TG (mmol/L)	0.0027	0.9500	2.2969	0.1356
TC (mmol/L)	0.0680	0.7925	2.2675	0.1236
Physical activity	3.8991*	0.04798	16.1207***	0.0001
Women				
BMI (kg/m^2^)	0.4690	0.4998	0.0122	0.9303
LBM (kg)	5.7822*	0.0212	0.0247	0.9198
SLM (kg)	5.7163*	0.0232	0.0269	0.9295
SMM (kg)	1.3956	0.2403	2.9600	0.0917
BFP (%)	4.9396*	0.0359	0.1451	0.7233
25(OH) D (ng/mL)	2.5642	0.1119	0.0205	0.9286
1,25(OH)2D (pg/mL)	0.6002	0.4500	0.8652	0.3695
Phosphorus (mmol/L)	0.0612	0.8106	0.5403	0.4308
Magnesium (mmol/L)	1.8836	0.1659	3.9301*	0.0415
TG (mmol/L)	0.0732	0.7820	2.0326	0.1649
TC (mmol/L)	0.4507	0.5167	0.5409	0.4733
Physical activity	0.4203	0.5221	1.4401	0.2375

Statistical significance was set at the levels of *p ≤ 0.05, **p ≤ 0.01 and ***p ≤ 0.001

**Fig. 1:**
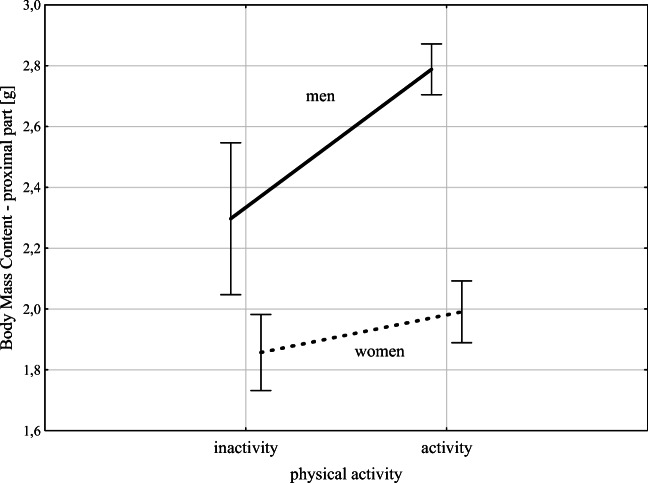
Relationship between body mass content in proximal part (g) among active and inactive men and women (results two-ways ANOVA analysis, *F* = 52,994; *p* = 0235), vertical lines-0.95 CI - confidence intervals

The strongest relationship between physical activity and bone mineral density parameters was found for BMC in men, especially for the proximal segment. In women, physical activity does not affect BMC values. Figure 1 presents the described relationships for men and women divided into inactive and active people (results of a two-factor ANOVA analysis of variance).

## Discussion

The aim of this cross-sectional study was to assess forearm bone tissue and its mineralisation level and bone mass in the group of young healthy women and men as well as analysing potential correlations between bone tissue status and modifiable factors connected with lifestyle (physical activity), biochemical indicators in blood and body composition. The study assessed the correlation of BMC and BMD with serum concentration levels of vitamin D, phosphorus, magnesium, total cholesterol and triglycerides. Moreover, the correlations between bone tissue status and LBM, SLM, SMM, BFP and physical activity were evaluated. Confounding variables were identified and controlled in the study.

The research results indicated correlations of BMD in the distal part in men with the level of TG and BFP. In turn, in the other part measured (the proximal part), BMD was affected by such variables as SMM, BFP, physical activity and serum 1,25(OH)2D concentration. Analogous analyses for women revealed a statistically significant correlation only between physical activity and BMD in the distal part. In the case of BMC, it was noted that, in men, BMC in the distal part correlated significantly with physical activity. Men who were physically active demonstrated significantly higher BMC in the distal part than physically inactive individuals. BMC in the proximal part in men correlated significantly with physical activity, SMM and BFP. Among young women, correlations between BMC and selected parameters turned out to be considerably weaker than in men. In the case of BMC in the distal part, significant correlations were revealed only for such body composition parameters as LBM, SLM and BFP. BMC in the proximal part in women correlated with magnesium concentration in serum.

There is a lot of evidence to support the thesis that physical activity (regular physical training in particular) leads to better BMD, especially in various parts of the skeleton in young men [26–28]. However, local influence of exercises is highlighted. In the locations where force is generated on a bone at the time of exercises, BMC and BMD are affected according to Wolff’s law [19].

Three studies revealed correlations between BMD of the femur neck and lower limb muscle strength [29–31]. In the study on young women and men from a sports university, only a strong correlation between active lifestyle and normal BMD in men was noted. Such a correlation was not revealed in women. A detailed mechanism of the effects of exercises on bone health has not been fully explained yet due to challenges connected with bone cell response in vivo. However, the influence of mechanotransduction on bones was particularly well described by Duncan and Turner [32]. Muscle contractures resulting from motor activity during physical exercises may evoke pressure of extracellular fluid on bone matrix and in this manner, they may affect the development of bone tissue. It is highlighted that gravity during training most likely exerts the largest influence when it comes to BMD increase [33]. Within their university curriculum, young men participating in the study perform activities connected with strength sports, team sports and combat sports, where forces affecting the musculoskeletal system are generated. Scientific research indicated that resistance training and weight-bearing activities may be particularly effective in maintaining and increasing BMD of the skeleton [34]. Unarguably, such a correlation is affected by muscle mass. A higher percentage of active tissues and SMM in general body mass in men may result in a better bone formation than in the case of women who have a thinner body build and more often take up activities with lower mechanical loads. In our research, a significant correlation between BMD and SMM was noted, particularly in the proximal part. In both men and women, a significant correlation between normal BMD in the proximal part and considerably higher SMM and LBM was revealed. This result is in line with the majority of studies in this area. It indicates a favourable influence of increased SMM on proper BMD values in various parts of the skeleton [35–37].

We also assessed the correlation between body fat and bone tissue parameters. Our study revealed no significant differences regarding BFP in the groups with proper and decreased bone mineralisation either in men or in women. Higher BFP did not lead to better BMD or BMC in young women and men. Correlations between BFP and BMD or BMC assessed in previous studies on various populations, particularly on women at menopause age and men at andropause age, have not been unanimous. In several observations, it was concluded that the risk of lower BMD and, therefore, of fracture at a later age decreases significantly together with BFP increase among women, but not among men. In turn, the conclusions of other authors showed that excessive body fat and BMI do not protect from BMD loss or osteoporosis occurring with age [38–40].

Our team also assessed the correlation between selected biochemical indicators in blood and mineral state of the forearm. In men, strong correlations were noted between serum 1,25(OH)2D and BMD in the proximal part as well as between triglyceride concentration and BMD in the distal part. In turn, in women, only magnesium concentration affected BMD in the proximal part. The research revealed that there are many nutrients and dietary elements such as macroelements, microelements or bioactive food which may potentially affect bone health. Assessment of the amount of active metabolites of macroelements, microelements and vitamins which are significant for bones may serve as an indicator of mineral status. Research results indicate a significant correlation between normal BMD in various parts of the skeleton and proper concentration levels of such markers as calcium; magnesium; phosphorus; sodium; potassium and vitamins A, D, E, K and C as well as macroelements such as protein and fatty acids [5].

Among numerous nutrients, the literature indicates a key role of vitamin D in the metabolism of bone tissue and its health. It was concluded that the main form of vitamin D in the bloodstream, i.e. 25-hydroxyvitamin D, was a substance with moderate biological (antirachitic) activity. In turn, 1,25-hydroxyvitamin D, which is created during hydroxylation, was concluded to be the most active form of this vitamin. In our research, it was the correlation between this form of vitamin D and BMD in the proximal part in men that could be defined as strong. In women, a significant correlation was noted only between magnesium concentration and BMC in the proximal part.

The study has some limitations. Certainly, the results relate to a small number of respondents and the test should be repeated in the future to try to extend for another group of young males and females. The project involved people living in a large urban area, so the results should be verified by research on males and females from smaller towns outside the central Poland. In addition, the relatively small sample size and the lack of longitudinal measurements limit the analysis to cross-sectional analyses. The lack of skeletal measures at other sites than the forearm is a limitation.

## Summary and conclusion

The level of physical activity significantly affected bone status, particularly among young men. The results of the study confirm the important role of physical activity for bone health. The results of the study indicate the need to monitor the state of bone tissue in a young population. Such observation will eliminate factors that reduce bone mass at a young age, and reduce the risk of osteoporosis at a later age. It is necessary to educate about the impact on healthy bones: proper body composition, especially good muscle mass and a good level of biochemical indicators, especially vitamin D.

In addition, the results should be interpreted with some trepidation. In over half of the young men, a deficit of vitamin D was noted. Apart from its basic effects on bone tissue health, this vitamin also serves other significant functions. In conclusion, further research is needed to determine exact causes of a high proportion of decreased mineralisation in the proximal part of the forearm among young people which was noted in the study. The role of screening among young people should be increased.
